# Reference-dependent preferences for maternity wards: an exploration of two reference points

**DOI:** 10.1080/21642850.2014.881257

**Published:** 2014-04-03

**Authors:** Einat Neuman

**Affiliations:** ^a^ Department of Economics, College of Management, Rabin Street, Rishon Le'Tzion, Israel

**Keywords:** preferences, maternity wards, discrete-choice-experiment, reference point, loss aversion, public health care, C25, I11, I18

## Abstract

It is now well established that a person's valuation of the benefit from an outcome of a decision is determined by the intrinsic “consumption utility” of the outcome itself and also by the relation of the outcome to some reference point. The most notable expression of such reference-dependent preferences is loss aversion. What precisely this reference point is, however, is less clear. This paper claims and provides empirical evidence for the existence of more than one reference point. Using a discrete choice experiment in the Israeli public health-care sector, within a sample of 219 women who had given birth, it is shown that respondents refer to *two reference points*: (i) a constant scenario that is used in the experiment; and (ii) also the actual state of the quantitative attributes of the service (number of beds in room of hospitalization; and travel time from residence to hospital). In line with the loss aversion theory, it is also shown that losses (vis-à-vis the constant scenario and vis-à-vis the actual state) accumulate and have reinforced effects, while gains do not.

## 1. Introduction and motivation

In conventional neoclassical consumer theory (usually referred to as the “Hicksian theory”), the individual decision-maker has a preference relation over bundles of consumption goods and services that satisfies certain axioms and can be represented by a utility function. It follows, that a person's valuation of the benefit from an outcome of a decision is determined solely by the intrinsic “consumption utility” of the outcome itself. However, much evidence has shown that humans are often more sensitive to how their current situation *differs from some reference level* than to the absolute level of the situation. Preferences are, therefore, conditioned by one's reference point. Accordingly, the benefit from an outcome is valued by its absolute level *combined* with its contrast to a reference point.

A notable expression of such reference-dependent preferences is loss aversion: individuals understand their options in decision-making problems as gains or losses relative to a reference point, which is normally the “current position” of the individual.^1^ The subjective valuation of a change in an endowment is generally greater when the deviation from the reference point represents a loss than when the same-sized change is perceived as a gain (Kahneman & Tversky, 1979; Thaler, 1980; Tversky & Kahneman 1991 – theory of reference-dependent preferences). Evidence for the effect of the reference point on preferences has been found in numerous studies related to various markets (see Neuman & Neuman, 2008, for a review and for an illustration based on the health sector).

What is the reference point used by an individual to evaluate preferences? The literature suggests: endowments (mainly when traded goods are considered); expectations (Koszegi & Rabin, 2006; Shalev, 2000); aspirations and goals (Koop & Johnson, 2012; van Osch, van den Hout, & Stiggel-bout, 2006 – suggesting multiple goals); and Neuman and Neuman (2008) who suggested that when a stated preferences technique was employed for preferences elicitation using a discrete choice experiment (DCE)^2^ with a constant scenario, then this arbitrary *constant scenario* served as the individual's reference point. Consequently, estimated preference patterns changed when the constant scenario was changed. In the same research, it was also shown that a negative deviation from the reference point (a loss) had a larger disutility than the utility of a same-sized positive deviation (a gain), thus supporting the loss aversion theory.

While apparently DCEs have not been previously employed for testing and evaluating reference-dependent loss aversion, they are highly suitable for such testing because they estimate the marginal valuations of attributes based on *deviations from a reference point* (a constant scenario).^3^ Moreover, reference-dependence can be *examined for each attribute separately*.

In the study reported in this article, it is suggested and demonstrated that *the individual is using more than one reference point in the process of evaluation of her preferences,* and accordingly, her choices. It is shown that even in stated preferences experiments, which are based on hypothetical scenarios, *the actual state apparently serves as an additional level of reference*. It follows that cognitive decision-making is most probably more complex than traditional economic models claim.

The next section briefly summarizes and extends the Neuman and Neuman's (2008) study, presenting evidence for use of the *actual state as an additional reference point* and lends *further support* to the loss aversion theory. The last section summarizes and concludes.

## 2. Reference-dependent behavior and loss aversion

### 2.1. A DCE with a constant scenario

In Neuman and Neuman (2008), reference-dependent preferences and the loss aversion theory were tested within the context of preference structures for maternity-ward attributes, estimated using data gathered from 1752 observations made by a sample of 219 women who had recently given birth in *three big public hospitals in the Greater Tel-Aviv area.* The subjects were approached by interviewers (during April–August 2003) and requested to fill out a questionnaire. In the questionnaire, a DCE was used to present individuals with a series of eight pairs of hypothetical scenarios (maternity wards), described in terms of a number of relevant multi-leveled attributes, varying from scenario to scenario. The respondents were also requested to report the *actual levels* of the two quantitative attributes “number of beds in room” and “travel time from residence to maternity ward”. Table 1 presents the five attributes included in the hypothetical scenarios and their levels (in the constant scenario A and in the varying scenario B), as well as the values of the actual measures of the two quantitative attributes.

**Table 1.** Attributes and levels.

Attributes	Constant ward A	All possible levels (ward B)	Actual level
Attitude of staff	Reasonable	Reasonable, very good	–
Professionalism of staff	Good	Good, very good	–
Information	Extensive	Basic, extensive	–
Number of beds in hospitalization room	2 beds	1, 2, 3 beds	1, 2, 3, 4, 5 (with respective frequencies of 1, 101, 109, 6 and 2)
Travel time to hospital	30 min	15, 30, 45 min	Range: 2–60 (mean = 17.38, SD = 10.6)

The data compiled from the completed questionnaires were used to estimate the basic preference structure (main effects) using random-effects probit regressions. The dependent variable is dichotomous and takes the value of 1 if maternity ward B was chosen and the value of 0 if maternity ward A was preferred. The independent variables represent the deviations of B from A. The qualitative attributes (“attitude”, “professionalism”, and “information”) were defined as dummy variables (=1 when B has the better level). The quantitative attributes (“number of beds” and “travel time”) that have three alternatives each, were also treated as dummy variables, where each three-level attribute was presented by two dummy variables. The regression coefficients represent relative valuations (utility scores) of the attributes.^4^

Number of beds was represented by the following two variables: (i) three beds = 1, if scenario B had three beds versus two in the constant A. Three beds represent a “loss”; and (ii) a private room = 1, if scenario B had a private room versus two beds in A. A private room represents a “gain”. Similarly, travel time was represented by the following two variables: (i) 45 min = 1, if travel time in scenario B was 45 min versus 30 min in the constant scenario A, representing a “loss”; and (ii) 15 min = 1, if travel time in scenario B was 15 min versus 30 in the constant scenario A, representing a “gain”.

The middle-level in the constant scenario A, facilitated testing for loss aversion for these two characteristics of the health service: individuals were loss-averse if the disutility of three beds (versus two beds in A) was larger than the utility associated with a private room (vis-à-vis two beds). Similarly, if travel time of 15 min more (45 min versus the constant scenario of 30 min) had a larger disutility than the utility related to travel time of 15 min (that is, 15 min less than the reference level), individuals were said to be loss-averse.

Regarding the three other attributes of maternity wards, the deviations B–A (A is the constant scenario), represent gains for “attitude” and “professionalism” and a loss for “information”. It follows that it is not possible to contrast the valuation of a “gain” against the valuation of a “loss”. In order to test the loss aversion hypothesis for these attributes too, an alternative set of questionnaires has been distributed with a different constant scenario – where the deviations from the constant scenario have the opposite directions.

Using the coefficients of preference structures estimated for data of the two sets of questionnaires, we were able to detect loss-averse behavior regarding four out of the five investigated maternity wards' attributes. The results were less conclusive for the “travel time from residence to hospital” attribute (see Neuman & Neuman, 2008, for details). In the study presented in this paper, we focus on the two quantitative attributes (for which we have also actual levels), and will therefore refer only to the first version of questionnaires.

### 2.2. Adding another reference point – the actual state

As pointed out, it is possible that behavior is more complex than assumed – that the individual *relates to more than one reference point* when expressing preferences and evaluating the utility of gains against the disutility of losses. For instance, within a DCE, which is a stated preferences technique, it is possible that the respondent relates also to the attributes' actual level as a *supplementary* reference point *in addition* to the constant scenario (A). This means that when the decision-making respondent considers whether to choose maternity ward B or not (prefer A), she bears in mind, simultaneously, both the deviation from the constant reference point (B–A), *as well as* the disparity between scenario B and the attribute's actual state (B – “actual state”). It therefore follows that ceteris-paribus (i) estimated preferences should differ if the actual state is different (even if the constant scenario is identical); and (ii) if the disutility of a loss is indeed valued more than the utility of a same-sized gain, we can expect that the additional decrease in utility caused by the perceived loss *vis-à-vis the actual state* will be larger than an *additional* increase in utility that results from a positive deviation from the actual level (a “gain”). Interaction terms between the relevant main effects (that relate to deviations of B from A) and deviations of B from the actual attribute levels, were introduced to test the effect of this potential *additional* reference point.

 Table 2 specifies eight combinations that relate to deviations from the two reference points and thus defines all possible gain/loss interactions.

**Table 2.** “Gains” and “losses” generated by deviations from the two reference points (level in constant A; and the actual level).

Case number	Level in B (dep. variable = 1 if B is preferred over A)	Level in A (constant scenario)	Actual level (generating a “gain” or a “loss” versus B)	Deviations when moving from A to B	Deviations when moving from the actual level to B
1	3 beds	2 beds	1 or 2 beds	Loss	Loss
2	3 beds	2 beds	4 or 5 beds	Loss	Gain
3	Private room (1 bed)	2 beds	2 beds or more (218 of 219 cases)	Gain	Gain (if applicable)
4	Private room (1 bed)	2 beds	Less than 1 bed (non-existent)	Gain	Loss (if existed)
5	Travel time of 45 min	30 min.	0–15 min	Loss	Loss
6	Travel time of 45 min	30 min.	Over 75 min (no observations)	Loss	Gain (if existed)
7	Travel time of 15 min	30 min.	45 min or more	Gain	Gain
8	Travel time of 15 min	30 min.	Negative travel time (non-existent)	Gain	Loss (if existed)

To define a loss/gain of travel time versus the actual travel time, we used a dummy variable that was set to 1 for a deviation (between B and the actual state) of at least 30 min. The rationale behind this definition was twofold: there were probably some measurement errors in the reported travel times because women on their way to give birth do not typically measure the exact travel time; and as travel to the hospital for giving birth is a one-time event, minor deviations are most probably not considered as gains/losses (this is one of the conclusions of the study reported in Neuman & Neuman, 2008).

As is evident from Table 2, the eight gain/loss combinations include four combinations that do not exist within our data (numbered in Table 2 as 3, 4, 6, 8). The other four combinations (numbered in Table 2 as 1, 2, 5, 7) resulted in four interaction terms that were added in to the basic main-effects regression in 2 Table 3.

**Table 3.** Main-effects regressions: women who had given birth – Israel, 2003.

Explanatory variables	Coefficients: basic model	Coefficients: extended model (including interactions with deviations from *actual* beds and travel time)
Attitude of staff	1.169 (14.69)	1.187 (14.73)
Professionalism of staff	1.165 (15.76)	1.183 (15.81)
Information	0.588 (8.48)	0.605 (8.64)
Number of beds (reference: 2 beds)		
Three beds (1 more = loss)	−0.711 (8.32)	−0.502 (4.88)
Private room (1 less = gain)	0.214 (2.23)	0.213 (2.23)
(Three beds = loss)*(actual 1 or 2 = loss)	–	−0.471 (4.09)
(Three beds = loss)*(actual 4 or 5 = gain)	–	0.099 (0.35)
Travel time to hospital (reference: 30 min)		
45 min (15 min more = loss)	−0.327 (4.13)	−0.116 (1.10)
15 min (15 min less = gain)	0.508 (4.89)	0.524 (4.97)
(45 min = loss)*(actual 0–15 = loss)	–	−0.363 (3.06)
(15 min = gain)*(actual over 45 = gain)	–	−1.022 (1.48)
Sample size	1752	1752
Number of women	219	219
Log Likelihood	−800.25	−785.11
*ρ* (rho)	0.182	0.174
*χ*^2^ to test rho = 0 (significance level)	32.36 (0.00)	28.72 (0.00)

Note: Stata 9 was used for estimation (random-effect probit with no constant). *Z*-statistics in parentheses.

The findings presented in Table 3 indicate that the actual state matters considerably.^5^ Moreover, additional evidence is present for the loss aversion theory:

loss vis-à-vis the actual state has also a major negative effect on utility, on top of the effect of loss versus the constant scenario. Gain has a much milder positive effect. Apparently, gain versus the actual situation does not have any significant additional effect.

Starting with the attribute “number of beds”: in the basic model, which does not relate to the actual number of beds,^6^ an additional bed (moving from two beds in A to three beds in B) resulted in a utility drop by a score of 0.711 (*Z* = 8.32, *p* = 0.000), whereas a removal of one bed (down to a private room) had a positive utility score which was more than three times smaller (score of 0.214; *Z* = 2.23, *p* = 0.000) – indicating the much more pronounced effect of a loss.

In the extended model, which includes also interactions with the actual state: having three beds (versus two beds in the reference set A), led to a decrease in the utility score by 0.502 (*Z* = 4.88, *p* = 0.000) when the actual state was also three beds. However, if the actual state was one or two beds, representing a loss in B *also* versus the actual state, there was an *additional decrease* in utility by a score of 0.471. The disutility therefore doubled, indicating that a negative deviation from A has a very similar effect to a negative deviation from the actual state. We observe *a loss upon loss, accumulating and reinforcing the “loss effect”, providing more evidence for the loss aversion theory*. On the other hand, *a gain upon the loss of three beds* (when the actual number of beds was four or five) *did not lead to any netting-out of the negative effect of the loss* (an insignificant interaction term, *Z* = 0.35, *p* = 0.725), again, giving more evidence to the differential effects of “losses” and “gains”.

Turning to the attribute of “travel time”: in the basic model, the effects of a loss (traveling 15 min more) and a gain (15 min less) are not significantly different (coefficients of −0.327 and 0.508). A chi square test for equality of (the absolute values of) these two coefficients, yielded a statistics of 1.39 (*p* = 0.238), probably because traveling to the maternity ward is a single short episode and as a result “losses” and “gains” of 15 min are similarly valued.

However, as is indicated by the extended model, losses (of 30 min or more) versus the real travel time to the maternity ward were more highly valued compared to same-sized gains vis-à-vis the real driving time. The insignificant coefficient of −0.116 (*Z* = 1.10, *p* = 0.273) relates to an increase in travel time from 30 to 45, when the actual state was more than 15 min. The interaction term, relating to a very short real travel time of 0–15 min, adds an additional pronounced loss vis-à-vis the actual time on top of the loss versus the constant scenario A, and results in a significant decrease in utility of 0.363 utility scores (*Z* = 3.06, *p* = 0.000), indicating the *negative significant impact of the pronounced loss versus the actual condition*. On the other hand, a *gain versus the actual state does not reinforce the gain vis-à-vis the constant A* (an insignificant interaction term; *Z* = 1.48, *p* = 0.138), lending more support to the much milder effect on utility that resulted from a gain.^7^ Moreover, while the basic model does not provide evidence for differential effects of “loss” and “gain” in travel time (versus a hypothetical constant scenario), we do find evidence in the interacted model for the stronger effect of a loss compared to a gain vis-à-vis the real travel time.^8^

## 3. Concluding remarks

The present paper, which builds upon Neuman and Neuman (2008), showed that decision-makers who were presented with pairs of hypothetical scenarios and requested to decide whether they preferred scenario B or A (a constant scenario), related to more than one reference point when they chose their preferred alternative. In addition to the “built-in” reference point (the constant set A), they also considered deviations from their actual state (actual number of beds in their hospitalization room and travel time from residence to hospital). These findings provide *additional empirical evidence* to (i) the relevance of reference point(s) in the determination of preferences; and to (ii) the behavioral loss aversion theory, which asserts that “losses” vis-à-vis a reference point(s) have a much more pronounced (negative) effect on utility than same-sized “gains”. Moreover, the findings suggest that the cognitive decision-making process is probably more complex than economists currently realize.

It follows that preference structures for a good/service that are elicited using DCEs (with a constant scenario) among users of this good/service (e.g. patients who use a health facility; residents who use a recreation site), should be treated with double caution: the estimated preference structure is affected not only by the constant scenario A (which is known and can be considered) but also by the actual levels of the attributes experienced by the respondents, which are usually unknown and cannot, therefore, be considered.^9^

A related question is the following: If users of a service relate to the actual level they experienced, what is the parallel reference point of non-users? If preference structures for maternity-ward attributes were estimated based on similar DCEs conducted among women who never gave birth, would they refer only to the constant scenario A? Or rather, would these respondents too have in mind some “actual” reference point (e.g. number of beds they saw when they visited a family member or friend who recently gave birth; or the average number of beds in a standard hospital). This question remains open for further research, preferably to be conducted in cooperation with cognitive psychologists and perhaps even brain scientists.

## Notes

1. Loss-aversion is also related to the “endowment effect” (identified by Thaler (1980); see also Knetsch (1989) and Kahneman et al. (1990)) and to the comparable phenomenon – “status-quo bias” – that holds in multiple-good choice problems (Samuelson & Zeckhauser, 1988). See Rabin (1998) for a review of literature on the “endowment effect” and “status-quo bias” and also Kahneman et al. (2000).

2. The DCE approach to preference elicitation is akin to the choice-based approach to consumer theory because it explicitly assumes that choices observed in DCEs “reveal the preferences” of individuals. Hypothetical alternatives offered in DCE surveys are constructed using experimental design theory and analyzed using random utility theory (McFadden, 1974).

3. It should be noted that the DCE technique does not require a constant scenario within the various pairs of scenarios that are presented to the respondent. There are other versions that do not include a constant set. See the textbook by Ryan et al. (2008) that describes the design and conduct of various versions of DCEs, combined with numerous research applications and methodological developments.

4. Trade-offs between attributes can be estimated by the ratio between the two relevant coefficients that relates to the marginal rate of substitution (MRS) between attributes. The MRS can be used to assess the relative importance of all attributes by calculating the extent to which each attribute is valued in terms of a numeraire attribute, e.g. time or monetary cost. In our study, it could be calculated how much more (in minutes) women were willing to spend traveling to the maternity in order to have more information transfer (extensive rather than good); a more professional staff (very good rather than good); and other attributes (not reported in the current paper). If we had had a delivery cost attribute, we could have estimated the willingness to pay for improvements in each of the other attributes.

5. The effects of the qualitative variables: “attitude of staff”, “professionalism of staff”, and “information transferred from staff to patient” (for which we do not have data on the actual states), have been discussed in detail in Neuman and Neuman (2008). It appears that attitude and professionalism have similar utilities and effects on preferences, which are about two times larger than the effect/utility of information transfer.

6. And therefore represents some weighted average of utility valuations based on the different real numbers of beds in hospitalization rooms (ranging from 1 to 5, in our sample).

7. The significant coefficient of 0.524 relates to a gain versus the constant scenario (30 min) when the real driving time was 45 min or less. The interaction term added an additional gain versus the actual time that was increased to more than 45 min. The insignificant interaction term indicates that an additional gain does not lead to more utility.

8. The differential effects of loss/gain versus the constant scenario and loss/gain vis-à-vis the real travel time could stem from the different sizes of the deviations. While a deviation of 15 min was used for B versus A, the deviation of B from the actual state was at least 30 min. It could well be that using a 30 min deviation also from B to constant A, would have resulted in a significantly stronger effect of the loss than the gain.

9. The actual state is relevant and most probably affects preference structures also in DCEs that do not use a constant scenario.
